# Low levels of *Methyl-CpG binding protein 2* are accompanied by an increased vulnerability to the negative outcomes of stress exposure during childhood in healthy women

**DOI:** 10.1038/s41398-022-02259-4

**Published:** 2022-12-08

**Authors:** Livia Cosentino, Francesca Zidda, Helene Dukal, Stephanie H. Witt, Bianca De Filippis, Herta Flor

**Affiliations:** 1 Center for Behavioral Sciences and Mental Health, Istituto Superiore di Sanità, Roma, Italy; 2 Institute of Cognitive and Clinical Neuroscience, Central Institute of Mental Health, Medical Faculty Mannheim, Heidelberg University, Mannheim, Germany; 3 Department of Genetic Epidemiology in Psychiatry, Central Institute of Mental Health, Medical Faculty Mannheim, Heidelberg University, Mannheim, Germany

**Keywords:** Predictive markers, Psychiatric disorders

## Abstract

Numerous mental illnesses arise following stressful events in vulnerable individuals, with females being generally more affected than males. Adverse childhood experiences are known to increase the risk of developing psychopathologies and DNA methylation was demonstrated to drive the long-lasting effects of early life stress and promote stress susceptibility. Methyl-CpG binding protein 2 (MECP2), an X-linked reader of the DNA methylome, is altered in many mental disorders of stress origin, suggesting MECP2 as a marker of stress susceptibility; previous works also suggest a link between MECP2 and early stress experiences. The present work explored whether a reduced expression of MECP2 is paralleled by an increased vulnerability to the negative outcomes of stress exposure during childhood. To this aim, blood *MECP2* mRNA levels were analyzed in 63 people without history of mental disorders and traits pertaining to depressive and anxiety symptom clusters were assessed as proxies of the vulnerability to develop stress-related disorders; stress exposure during childhood was also evaluated. Using structural equation modeling, we demonstrate that reduced *MECP2* expression is accompanied by symptoms of anxiety/depression in association with exposure to stress in early life, selectively in healthy women. These results suggest a gender-specific involvement of *MECP2* in the maladaptive outcomes of childhood adversities, and shed new light on the complex biology underlying gender bias in stress susceptibility.

## Introduction

Mental disorders are a serious public health issue with highly debilitating outcomes that have a significant impact on both affected individuals and society [1]. The burden associated with mental illnesses is considerable: according to the World Health Organization, in 2019 one in every eight people around the world were living with a mental disorder such as anxiety-, mood- or trauma-related disorders [2]. Access to quality mental health care and effective treatment is limited, with a substantial gap between people in need and those receiving mental health services, leading to disability and premature death due to preventable physical conditions or suicide [3].

Stressful life events clearly precede the onset of many episodes of depression and anxiety, and post-traumatic stress is known to be triggered by traumatic experiences [4, 5]. However, stress/trauma per se is not sufficient to account for the occurrence of psychopathology, and the probability to develop disorders of stress origin following adverse experiences relies on individual vulnerability [6]. Although the factors underlying interindividual differences in stress susceptibility have not yet been completely clarified, the overall disease risk profile appears to depend on the interaction among genes and environmental factors [7–9], which eventually shapes the way individuals respond to stress [6]. The inability to adaptively face stressors in fact clearly determines an higher incidence of psychopathology [10–12].

Epigenetic regulation of gene transcription is a mechanism by which the gene × environment interaction occurs. Epigenetics in fact provides a mechanism to translate environmental exposures into the modulation of gene expression, thus altering stress adaptability and influencing the probability that an individual will display susceptibility or resilience to future stressors [6].

Among the many possible epigenetic modifications, DNA methylation has been largely studied due to its extreme responsiveness to environmental stimuli. Indeed, the methylation status of specific loci appears to quantify the amount of stressors experienced throughout life [13–15]. Such a dynamic nature makes it a promising mediator of behavioral adaptations to environmental challenges, suggesting that it can prompt individuals to risk or resilience beyond the variability attributable to genetic factors alone. Of note, risk and resilience have been proposed to associate with an opposite methylation profile on similar genes, consistent with the idea that a disrupted balance between activation and repression of gene expression may interfere with the ability to adaptively respond to stress [15, 16].

The X-linked Methyl-CpG binding protein 2 (MECP2) is a reader of the DNA methylome and plays a major role in the regulation of gene expression in the brain [17]. MECP2 activity, which is regulated by post-translational modifications of the protein, is modulated by neuronal firing, thus strongly contributing to the adaptability of neurons to a dynamic environment [18]. Of note, stressful events early in infancy, which are recognized to be a major cause of increased vulnerability to future challenges [19], were found to affect MECP2 functionality, thus allowing an enduring reorganization of methylation and expression of stress-related genes [20], suggesting a potential involvement of this protein in shaping vulnerability to develop stress-related psychopathology [21]. In line with this, *MECP2* was found to be mutated or differentially expressed in a number of mental disorders whose onset can be triggered by stress such as schizophrenia, bipolar disorder and depression [22–24]. Moreover, regulation of MECP2 functionality has been suggested to contribute to both depressive-like symptoms and their mitigation by selective serotonin reuptake inhibitors, as well as to drug craving in substance abuse disorders [25–27].

In spite of the increasing evidence of MECP2 involvement in stress-related psychopathology, its role in prompting disease vulnerability has so far received little attention. We have shown that a hypomorphic mutation in the *MeCP2* gene provides vulnerability to develop behavioral and molecular features of post-traumatic stress disorder (PTSD) in trauma-exposed mice, suggesting that MECP2 could represent a marker of stress susceptibility [28, 29].

Based on this evidence, this study explored whether *MECP2* expression levels may be associated with increased stress vulnerability in humans. To this aim we examined *MECP2* expression levels in blood samples from healthy volunteers, and assessed *MECP2* interaction with depressive and anxiety symptom clusters, hereby considered as proxies of increased risk to develop mental disorders related to stress. Previous studies have in fact demonstrated that individuals with subthreshold symptoms are more likely to develop a mental disorder compared to individuals without such symptoms [30, 31].

Given the reported connection between MECP2 function and stress early in life [21], and the widely recognized influence that the latter exerts in increasing the risk of adult psychopathology [32], we explored the possibility that childhood adversities play a fundamental role in the association between blood *MECP2* levels and vulnerability to stress-related psychopathology. Therefore, we hypothesized that reduced *MECP2* expression may specifically mark the increase in anxiety/depression symptoms associated with stress exposure during infancy or adolescence.

Since women are known to be more prone to develop mood- and stress-related illnesses following abuse or neglect in childhood, and MECP2 function has been linked to the establishment of developmental sex differences in mouse models [33–35], we assumed that the hypothesized connection between reduced levels of *MECP2* and the maladaptive outcomes of early adverse experiences might be stronger among women, and thus examined the role of gender in this correlational model. Present findings underline the role of MECP2 in the risk for psychopathology and strengthen our knowledge on the gender-specific biology beneath stress vulnerability.

## Materials and methods

### Study participants

Sixty-three healthy volunteers of Caucasian ethnicity (23 females; mean age: 36.79, standard deviation: 15.78, range: 18–69 years of age) participated in the study (see supplementary information for further details). The sample considered in the present study overlaps with that of previous work [36, 37]. Individuals with current or lifetime mental disorders such as major depressive disorder, current or chronic substance abuse, schizophrenia or borderline personality disorder, as assessed with the Structured Clinical Interview for the Diagnostic and Statistical Manual of Mental Disorders-IV [38, 39], were excluded. Written informed consent was obtained before the experiment, which was approved by the Ethics Committee of the Medical Faculty Mannheim, Heidelberg University. The study conformed to the Code of Ethics of the World Medical Association (Declaration of Helsinki, 6th revision, 2008).

### MECP2 expression

Whole blood was collected in PAXgene Blood RNA (PreAnalytiX) or Tempus Blood RNA (Applied Biosystems) Tubes (*N* = 16 and 47, respectively) [40]. Total RNA including miRNA was extracted using the PAXgene Blood miRNA Kit (Qiagen), or the Tempus™ Spin RNA Isolation Kit (Applied Biosystems), respectively, following the manufacturer´s protocols. The concentration of the RNA samples and the sample purity was assessed with NanoDrop 1000 Spectralphotometer (Thermo Scientific). The cDNA was synthesized by a reversed transcription reaction using the High Capacity cDNA Reverse Transcription Kit (Applied Biosystems). Quantitative PCR was performed on the QuantStudio 7 Flex Real-Time PCR System (Applied Biosystems by Life Technologies) by using TaqMan Fast Advanced Mastermix (Applied Biosystems), and the *MECP2* TaqMan Gene Expression Assay Hs00172845_m1 (Applied Biosystems). The *Actin Beta* (*ACTB*) TaqMan Gene Expression Assay Hs01060665_g1 (Applied Biosystems) was used as an internal standard. Results were calculated with the QuantStudio Real-Time PCR Software v1.3 (Applied Biosystems by Thermo Fisher Scientific) by the comparative 2^−ΔΔCt^ method and normalized to *ACTB*. Analyses were carried out in triplicates. An unpaired *t*-test revealed that blood tubes did not significantly influence *MECP2* levels (t_61_ = −0.534, *p* = 0.595).

### Psychometric measures

Depression and anxiety measures have been selected in representation of stress-related symptom clusters. Trait anxiety was measured with the German version of the Trait scale of the State-Trait Anxiety Inventory (STAI-T; [41]), and depressive symptoms were assessed by administering the German version of the Center for Epidemiological Studies Depression Scale (CES-D; [42]). The anxiety and depression scales of the German revised version of the Symptom Checklist-90 (SCL-90-R [43]), a self-report instrument evaluating a broad range of psychological problems, were also used for validation. Importantly, these scales are all psychometrically validated (see supplementary methods) and designed to be subjected to clinical and non-clinical (healthy) populations, mainly for research purposes or as a screening tool to identify people who may be diagnosed with a mental disorder [44, 45].

Participants’ recall of adverse experiences during childhood was assessed using the German version of the Childhood Trauma Questionnaire (CTQ; [46]), a broadly used retrospective self-report instrument aimed at quantifying the severity of emotional/physical abuse and neglect, and sexual abuse experienced up to 18 years of age. Current exposure to chronic stress was evaluated with the Trier Inventory for Chronic Stress (TICS; [47]), aimed at measuring the presence of chronic stressors in everyday life in terms of their intensity, duration and frequency (see supplementary methods).

### Statistical analyses

All statistical analyses were conducted using SPSS 20.0 and AMOS 20.0 (IBM Statistics).

All data were normally distributed, or transformed, to ensure normal distribution (see supplementary information, Table S1); equality of variances was tested by means of Levène test. Unpaired *t*-tests and Pearson correlations were used to evaluate whether *MECP2* expression differed between genders or changed with age, and the effect sizes were calculated according to Cohen [48]. Multiple regression analyses (95% confidence intervals) were used to evaluate the main and interaction effects of *MECP2* expression and gender (females = 1, males = 2) on the severity of childhood adversities and of stress-related symptomatology (depressive and anxiety symptoms). In addition, regression analyses (95% confidence intervals) were used to evaluate the direct relationships between childhood adversities and a symptom profile highly associated with risk of stress-related psychopathology.

Structural Equation Modeling (SEM) with maximum likelihood estimation was then employed to test the prediction that *MECP2* expression marks the increased vulnerability to stress associated with early life stress exposure in a gender-dependent manner. Since the parameters included in the model were measured at the same time, the tested hypothesis is purely correlational.

Exclusion criteria for the model were: failure to converge after 240 iterations, the presence of squared multiple correlation values greater than 1 (R^2^ > 1) and poor fit, estimated via the following goodness-of-fit measures: the χ^2^ statistic (with a good fit indicated by χ^2^/degrees of freedom (df) < 3), the root mean square error of approximation (RMSEA, with a good fit indicated by an index <0.08), the comparative fit index (CFI, with a good fit indicated by an index >0.95), the Tucker-Lewis Index (TLI, > 0.95 indicating acceptable fit) and the Standardized Root-Mean-Square Residual (SRMR, acceptable fit if <0.10) [49, 50]. When the overall model fit was poor, model respecifications were made by removing nonsignificant directed arcs (*p* > 0.05) and adding correlated paths as indicated by modification indices that were consistent with the hypothesis. The statistically significant improvements between hierarchical nested models could be assessed using the likelihood ratio (calculated as the difference in χ^2^ and df between the models of interest [51]). To establish mediation, indirect paths were tested for significance using a Bias-Corrected (BC) Bootstrapping method (95% confidence intervals; 2000 resamples [52]).

To examine whether the final model was specific for early life stressful experiences, it was retested with a measure of current perceived chronic stress replacing the childhood trauma scale. Also, we checked whether the final model predicted equally well anxiety and depressive symptoms when using different symptoms scales established in the literature. All tested models complied with the rule of including at least 10 observations per indicator variable [53]. For each of the analyses the alpha level was set to 0.05 [51, 54].

## Results

### *MECP2* is overexpressed in females compared to males

**Fig. 1:**
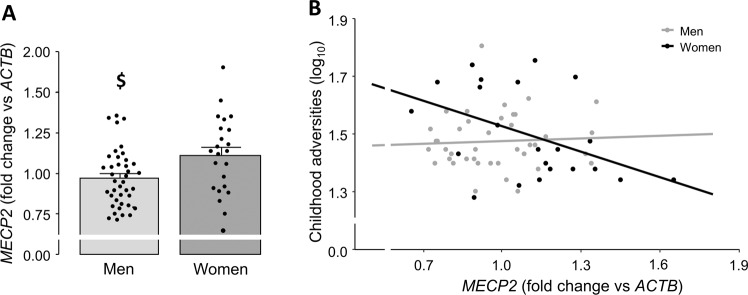
*MECP2* is overexpressed in women compared to men and gender moderates *MECP2* association with childhood adversities. **A** Blood mRNA levels of *methyl-CpG binding protein 2* (*MECP2*) are increased in women compared to men. Statistical significance was calculated by the means of non-directional Student’s *t*-test. ^$^*p* < 0.05. Data are Mean ± standard error of the mean (SE). **B** Decreased expression of *MECP2* is associated to more severe childhood adverse experiences, particularly in women. Statistical significance was calculated by the means of multiple linear regression. R^2^: 0.206 (Women); 0.003 (Men).

We tested whether gender influenced the expression levels of *MECP2*, an X-linked gene, in the blood of participants. Interestingly, we found that *MECP2* mRNA was significantly higher in women compared to men (t_61_ = 2.689, *p* = 0.009, Cohen’s d = 0.704; Fig. 1A), which underscores the existence of gender differences in the regulation of *MECP2* expression. *MECP2* levels did not significantly correlate with participants’ age (r_61_ = −0.155, *p* = 0.224), thus excluding the possibility that differences in the age of the subjects might have confounded the results.

### Low *MECP2* levels are associated with severe childhood adversities especially in females

To test the hypothesis that *MECP2* expression might be directly associated with stress-related measures in a gender-dependent manner, multiple regression analyses were performed. No significant main or interaction effects of *MECP2* and gender were found for the association with depressive or anxiety symptoms (Table 1). By contrast, growing up in an adverse environment was significantly related to higher levels of depressive symptoms (β = 0.271, *p* = 0.032) and trait anxiety (β = 0.373, *p* = 0.003), which reaffirms the existence of a link between exposure to early adversities and lasting vulnerability to stressors. See Table 1 for further details.

**Table 1:** Results of multiple regression analyses.

Regression models	b ± SE	CI	β	t	*p*-value
*MECP2* + gender + MECP2*gender → childhood adversities					
* MECP2*	−0.019 ± 0.016	−0.051 to 0.014	−0.153	−1.144	0.257
gender	−0.021 ± 0.016	−0.052 to 0.010	−0.175	−1.356	0.180
MECP2*gender	0.033 ± 0.015	0.003 to 0.064	0.285	2.217	0.030
*MECP2* + *gender* + MECP2*gender → depressive symptoms					
* MECP2*	−0.036 ± 0.045	−0.126 to 0.054	−0.114	−0.805	0.424
gender	−0.030 ± 0.043	−0.117 to 0.057	−0.094	−0.692	0.492
MECP2*gender	0.031 ± 0.042	−0.053 to 0.115	0.101	0.742	0.461
*MECP2* + gender + MECP2*gender → anxiety symptoms					
* MECP2*	0.001 ± 0.016	−0.032 to 0.033	0.006	0.046	0.964
gender	−0.014 ± 0.016	−0.046 to 0.017	−0.124	−0.912	0.365
MECP2*gender	0.016 ± 0.015	−0.014 to 0.047	0.145	1.071	0.288
*Childhood trauma* → depressive symptoms	0.712 ± 0.324	0.064 to 1.360	0.271	2.196	0.032
*Childhood trauma* → anxiety symptoms	0.355 ± 0.113	0.129 to 0.581	0.373	3.142	0.003

*Abbreviations:* *MECP2* - methyl-CpG binding protein 2, *b -* unstandardized coefficient, *SE* - standard error, *CI* - 95% confidence intervals for b, *β* - standardized coefficient, *t* - Student’s *t* statistic. Symbols: underlined—significant results.

Of note, neither *MECP2* nor gender alone were directly associated with the severity of childhood aversive experiences. Interestingly, however, gender moderated *MECP2* association with childhood adversities (MECP2*gender—β = 0.285, *p* = 0.030). In particular, lower peripheral *MECP2* expression levels were related to higher childhood adversity scores especially in female participants (Fig. 1B), further corroborating the existence of a gender-dependent association between MECP2 and stress at infancy.

### Reduced *MECP2* expression is linked with the negative outcomes of exposure to childhood adversities

**Fig. 2:**
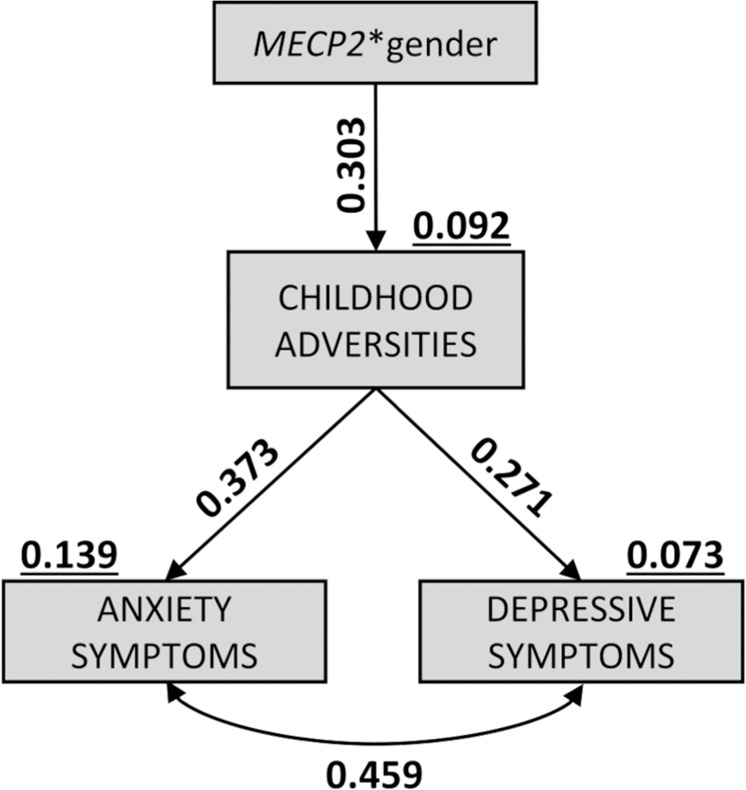
Reduced *MECP2* expression is linked with the increase in anxiety/depression symptoms associated with exposure to childhood adversities in healthy women. Expression of *methyl-CpG binding protein 2* (*MECP2*) interacts with gender in associating with the severity of childhood adversities. This, in turn, mediates an indirect relationship with depressed mood and trait anxiety. Statistical significance was calculated by the means of Structural Equation Modeling (SEM) with maximum likelihood estimation and Bias-Corrected Bootstrapping method. [Symbols: → directed arcs (*p* < 0.05); ↔ correlations (*p* < 0.05); black numbers—standardized coefficients; black underlined numbers—explained variance (R^2^)].

The SEM analysis focused on the hypothesis that reduced *MECP2* expression may specifically mark the increase in anxiety/depression symptoms associated with stress exposure during infancy or adolescence. In particular, in the initial base model (Table 2, 1i and Fig. S1A), we tested the hypothesis that gender moderates *MECP2* prediction of stress-related symptoms via childhood adversities. The second model (Table 2, 1ii and Fig. S1B) used modification indices to test the addition of four paths to the initial model. Three of these paths included correlations: one between the symptoms of stress-related disorders (anxiety and depression) and two between the input variables (*MECP2* and gender with the interaction term MECP2*gender), while the fourth path was a direct arc from gender to *MECP2*. The addition of these four paths significantly improved the model fit (significant likelihood ratio, *p* < 0.001). In the final model (Table 2, 1iii and Fig. 2) remaining nonsignificant paths were removed, and the model fit was further, although not significantly, improved. The overall model fit of this final model was satisfactory (Table 2).

**Table 2:** Goodness of fit indices.

Model	*Ν*	χ^2^	df	χ^2^/df	Δχ^2^	Δdf	RMSEA	SRMR	TLI	CFI
(1) Hypothesized *MECP2* model										
(i) Base model	63	30.108***	10	3.101	—	—	0.180	0.143	0.198	0.465
(ii) Four paths added	63	1.559	6	0.260	28,549***	4	0	0.028	1.295	1
(iii) Final model: nonsignificant paths removed	63	0.114	2	0.057	1445	4	0	0.012	1.197	1
(2) Confirmatory models										
(iv) Chronic stress model	62	0.669	2	0.335	—	—	0	0.033	1.094	1
(v) Depression/anxiety scales substitution model	63	0.674	2	0.337	—	—	0	0.013	1.050	1

*Abbreviations: MECP2* - methyl-CpG binding protein 2, *N -* sample size, *χ*^*2*^ - chi square statistic, *df* - degrees of freedom, Δ*χ*^*2*^ - nested chi square difference, *RMSEA* - root mean square error of approximation, *SRMR* - standardized root mean square residual, *TLI* - Tucker-Lewis index, *CFI* - comparative fit index. Symbols: * - *p* < 0.05; *** - *p* < 0.001.

In terms of variance, the final model accounted for 13.9% of the variance in anxiety symptoms and 7.3% of depressive symptoms, and these symptoms were predicted by the effects of the MECP2*gender interaction on childhood adversities (9.2%), suggesting that the relationship between childhood adversities and a symptom profile highly associated with risk of developing stress-related psychopathology is differentially marked by *MECP2* in men and women.

In the final model gender was found to moderate the association between *MECP2* expression and increased severity of childhood adversities (MECP2*gender—β = 0.303, *p* = 0.012). Worse experiences during childhood were associated with greater depressed mood (β = 0.271, *p* = 0.027) and increased trait anxiety (β = 0.373, *p* = 0.002). Depression and anxiety were positively correlated (r = 0.459, *p* < 0.001). See Table S2 for further details.

Bootstrapping confirmed a significant indirect pathway from MECP2*gender down to symptom outcomes, indicating that childhood adversities significantly mediated the association between *MECP2* expression and symptomatology typical of stress-related disorders in a gender-dependent manner (Table 3).

**Table 3:** Indirect effects in the final and confirmatory models.

Total indirect pathway	b ± SE	CI	β	*p*-value
MECP2*gender → depressive symptoms				
Final model	0.025 ± 0.015	0.002 to 0.063	0.113	0.029
Chronic stress model	0.014 ± 0.018	−0.016 to 0.055	0.044	0.315
Depression/anxiety scales substitution model	0.011 ± 0.007	0.002 to 0.033	0.091	0.017
MECP2*gender → anxiety symptoms				
Final model	0.013 ± 0.007	0.002 to 0.029	0.082	0.012
Chronic stress model	0.007 ± 0.009	−0.009 to 0.024	0.061	0.368
Depression/anxiety scales substitution model	0.005 ± 0.004	0.000 to 0.016	0.064	0.043

*Abbreviations: MECP2* - methyl-CpG binding protein 2, *b* - unstandardized coefficient, *SE* - standard error, *CI* - 95% confidence intervals for b, *β* - standardized coefficient. Symbols: underlined—significant results.

### *MECP2* is not associated with the increase in stress-related symptomatology due to current stress exposure

To examine whether *MECP2* association with symptoms of depression and anxiety was specifically mediated by stress experienced during childhood, the final model was retested with current chronic stress load (TICS) replacing childhood adversities (Chronic stress model). The goodness of fit (GOF) indices of this confirmatory model matched the criteria for model acceptance (Table 2, 2v), thus allowing the interpretation of model paths. As expected, chronic stress was significantly associated with stress-related symptomatology (depression—β = 0.426, *p* < 0.001; anxiety—β = 0.588, *p* < 0.001), leading to a high percentage of variance explained for both depression and anxiety symptoms (18.1% and 34.6%, respectively). However, current stress load was not significantly predicted by the MECP2*gender interaction (Table S2). This resulted in a lack of significance of the indirect effects of MECP2*gender on symptom outcomes (Table 3), which suggests that remote, but not current stressful experiences, selectively mediate the gender-moderated association of *MECP2* with stress-related symptomatology.

### The gender-specific association between *MECP2* and early life stress-dependent vulnerability is confirmed using different psychometric scales

A third confirmatory model aimed at validating the strength of the final model by retesting the same path with the use of different scales measuring the severity of depression and anxiety symptoms. The GOF indices were acceptable (Table 2, 2vi). In terms of variance accounted for, the model was able to explain 4.4% of the variance for anxiety and 9% for depressive symptoms.

As expected, gender moderated the association between *MECP2* expression and childhood adversities (β = 0.303, *p* = 0.012). Worse childhood experiences, in turn, were associated with more severe stress-related symptomatology, although the association with anxiety missed significance (depression—β = 0.300, *p* = 0.013; anxiety—β = 0.210, *p* = 0.090). Importantly, the indirect effect of the MECP2*gender interaction on symptoms of anxiety and depression remained significant (Table 3), confirming the gender-dependent mediating effect of childhood adversities on the association between *MECP2* expression and stress-related symptoms over different psychometric scales. By generalizing our findings to different measures of depression and anxiety, this result further strengthens the hypothesized link between *MECP2* peripheral expression and the maladaptive outcomes of exposure to early life adversities in healthy women.

## Discussion

The translation of stressful experiences into mental disorders appears to be highly dependent on each subject’s vulnerability. There is a strong interest in determining the biological underpinnings of such interindividual differences in stress responses and outcomes, which would be helpful for identifying measurable markers of stress vulnerability and targets to increase resilience. The present study provides novel evidence that, in healthy people, reduced peripheral expression of *MECP2* is linked with reports of adverse childhood experiences, and with the associated increase in anxiety and depression levels. Since depression and anxiety traits are key factors boosting the risk of developing stress-related psychopathology, the present findings corroborate the hypothesized link between *MECP2* levels and stress susceptibility. Importantly, the reported effects were all moderated by gender, suggesting that the role of *MECP2* in the regulation of stress vulnerability differs between men and women.

The present results support the involvement of *MECP2* in shaping vulnerability to psychopathology. This is sustained by multiple evidence of an association between *MECP2* alterations and several mental disorders, including depression, bipolar disorder, schizophrenia and substance abuse [22–24, 26, 55]. Evidence of MECP2 involvement in such a large spectrum of disorders, whose major common factor is the etiological component of stress, led us to postulate a role for MECP2 in shaping stress susceptibility. The present study extends the connection between stress and *MECP2* to healthy conditions in a normative sample, and provides evidence that reduced *MECP2* expression is associated with proxies of increased vulnerability to stressors especially in women.

Of note, previous studies on rodents outlined the involvement of MECP2 in the establishment of lasting neuroendocrine and behavioral alterations following early life challenges [20, 21]. In line with this, we show that the link between *MECP2* and vulnerability to psychopathology is significantly influenced by exposure to adversities during childhood. Indeed, in accordance with previous findings [56–58], the severity of stressors experienced before age 18 correlates similarly with depressed mood and trait anxiety across psychometric scales. Notably, the subjects included in the present study reported early adverse experiences that do not meet the definition of traumatic; the fact that these experiences, mainly emotional abuse and neglect, hold lasting negative outcomes is in line with the clinical literature that supports a cumulative model of stress effects on vulnerability to psychopathology [59, 60].

Importantly, in this work we delineate that the increase in stress-related symptoms associated with early adversities is specifically linked with *MECP2* downregulation. In fact, while current stress load also predicts higher levels of depression and anxiety, ongoing stressors fail to mediate the association between stress-related symptoms and *MECP2* expression. Consistent with this, reduced levels of *MECP2* mRNA in the blood of participants are associated with the reported severity of early, but not current, adverse experiences.

This selectivity may reside in the cognitive effort required when people are asked to recall events from their childhood, which is not necessary while reporting ongoing circumstances. In fact, the memory mechanisms engaged by the first, but not the second task are likely to be modulated by MECP2, whose role in learning and memory processes is widely recognized [61]. In this line, reduced *MECP2* levels might come together with a blurred and biased recall of remote, but not current, experiences. However, there might also be a direct link between *MECP2* levels and early stressors. Rodent studies previously demonstrated the occurrence of an interplay between MECP2 and stress exposure during infancy [20, 62–64], picturing MECP2 alterations as a consequence of exposure to early stressors. Indeed, early life adversities were found to induce a persistent modulation of either MECP2 functionality or expression in the brain of rodent models [21, 65, 66]. Prospective studies on human cohorts are needed to ultimately shed light on the nature of the interplay between MECP2 and stress exposure during childhood.

Interestingly, gender turned out to play a major role in this scenario. Indeed, *MECP2* association with childhood adversities as well as its indirect link with stress-related symptoms, were moderated by gender. In particular, decreased *MECP2* expression was associated with more severe childhood experiences especially in females, suggesting a gender-specific involvement of MECP2 in supporting stress vulnerability. In this line, gender was previously found to moderate the detrimental consequences of childhood traumas, with girls reporting histories of abuse or neglect in adolescence displaying more severe depressive symptoms than boys [67, 68]. The gender-dependent association between *MECP2* levels and early adversities demonstrated in this study might provide a biological framework for the differential vulnerability to childhood traumas outlined in females and males. In this line, gender differences in DNAm, the epigenetic mark targeted by MECP2, have been previously suggested to play a role in the establishment of distinct thresholds of resilience to traumas in males and females [69, 70]. These differences probably derive from the permanent effects that sex hormones exert on the epigenetic machinery early in development (organizational effects) [71, 72]. However, as stated above, we cannot exclude that childhood adversities might have, themselves, gender-dependently altered *MECP2* expression. Indeed, sex differences in the alteration of MECP2 expression upon early stress exposure have already been shown in rodent studies, although the relative direction of mRNA or protein modulation was inconsistent [66, 73, 74]. Different type and timing of the stressors might have played a role in the lack of uniformity across studies, and further research is needed to clarify and dissect the nature of the correlation between *MECP2* expression and early life stress.

It is important to note that we did find significant differences in *MECP2* mRNA levels in the blood of male and female participants, with women overexpressing *MECP2* compared to men. Although *MECP2* is not expected to escape X inactivation outside embryonic development [75, 76], multiple mechanisms, involving hormonal or genetic factors in interaction with age and tissue specificity, have been proposed to account for differences in the expression of X-linked genes between males and females [77, 78] and may be involved in the gender-dependent regulation of blood *MECP2* mRNA outlined in the present work. Importantly, perinatal differences in *MECP2* expression between male and female rodent brains were previously described to depend on the specific region investigated [78, 79], corroborating the relevance of a tissue-dependent regulation. A gender-specific modulation of *MECP2* expression in the postnatal brain is also sustained by the sex-dependent effects of MECP2-associated neurological disorders, with *MECP2* loss of function (Rett syndrome) and duplication syndromes affecting primarily girls and boys, respectively [80]. In accordance with this, we report that women expressing lower levels of *MECP2* are particularly vulnerable to suffer from symptoms of depression and anxiety in association with childhood adverse experiences. These findings are also in line with the fact that girls are known to be more affected than boys by the detrimental effects of stress exposure throughout puberty, leading to a higher risk of developing mood- and stress-related disorders [33].

It is important to consider that the present findings have been obtained in healthy participants. This condition allowed us to assess significant associations between *MECP2* expression and subthreshold symptoms of depression and anxiety, known to be related to an increased risk of developing psychopathology. Although the present results are limited by their correlational nature, which does not allow us to derive conclusions on the predictive role of decreased *MECP2* expression with respect to prospective disease development, they may help in uncovering an innovative marker of vulnerability. Highly discriminative measures able to characterize vulnerable individuals are in fact still missing, and the multiplicity of factors involved in vulnerability to psychopathology challenges the finding of a common framework, which is needed to develop a predictive susceptibility index. So far the most promising attempts leverage merging candidate biological markers of stress susceptibility to construct integrative vulnerability measures [81]. Further research aimed at outlining novel promising markers, including epigenetic modulators, is thus encouraged.

Notably, since transcriptional profiles can be tissue-specific, peripheral *MECP2* downregulation may not reflect a similar modulation within the brain, the organ where MECP2 plays its most important function, which restricts possible interpretations on the central mechanisms involved in disease risk. While further studies are certainly needed to unravel what happens to *MECP2* within the brain, the relevance of its peripheral alterations for brain-related traits is encouraged by previous work that demonstrated associations between peripheral and central stress-induced epigenetic responses at MECP2-targeted loci [9, 70], and overlaps in blood and brain transcriptomic profiles in *MeCP2*-mutated mice [82].

In line with recent evidence on the contribution of X-linked genes to the existing gender bias in multiple mental illnesses [83, 84], the present results suggest an involvement of *MECP2* in providing vulnerability to stress-related psychopathologies, especially in females. Due to the small sample size, however, the results have to be interpreted with caution and need to be replicated in larger groups. Also, to verify whether such associations can be predictive of psychopathology itself, future large-scale longitudinal studies will have to be performed that may help in assessing whether decreased *MECP2* expression in healthy individuals prospectively predicts disease onset. Longitudinal evaluations might also strengthen the interpretation of the role played by childhood adversities, since these data are based on a retrospective evaluation and might be influenced by biases related to vulnerability to stressors.

Overall, the present results suggest a gender-specific involvement of low peripheral *MECP2* levels in supporting vulnerability to psychopathology when persons are facing adversities during childhood. Further studies are necessary to longitudinally confirm the proposed association, by monitoring if *MECP2* levels in health actually predict the likelihood of disease onset, thus representing a measurable marker of increased susceptibility for developing mental illnesses. The present findings shed new light on the complex biology underlying stress vulnerability and provide a novel promising candidate vulnerability marker to be further explored.

## Supplementary information

Supplement

## Data Availability

Ethical restrictions to protect participant confidentiality prevent us from making anonymised study data publicly available. Readers seeking access to the study data and materials should contact the corresponding author based on a formal collaboration agreement. This formal collaboration agreement indicates that data will be shared with other researchers who agree to work with the authors, and for the sole purpose of verifying the claims in the paper. The data and materials will be released to requestors after approval of this formal collaboration agreement by the local Ethics Committee of the Medical Faculty Mannheim.
