# Supplementary material for: Low levels of Methyl-CpG binding protein 2 are accompanied by an increased vulnerability to the negative outcomes of stress exposure during childhood in healthy women
Source: Transl Psychiatry. 2022 Dec 8;12:506. doi: 10.1038/s41398-022-02259-4 (PMC9731965; doi:10.1038/s41398-022-02259-4)
Supplement: Supplementary file 1 — Supplement [file 41398_2022_2259_MOESM1_ESM.docx]

**Supplement to:**

**Low levels of Methyl-CpG binding protein 2 are accompanied by an increased vulnerability to the negative outcomes of stress exposure during childhood in healthy women**

**Supplementary methods**

**Study Participants**

Forty-seven out of 63 participants were recruited via the central registry of the city of Mannheim (Germany) to take part to cross-sectional studies on learning and brain plasticity in post-traumatic stress disorder (PTSD); the remaining 16 participants are instead part of an ongoing prospective study on determinants of risk for PTSD, and were recruited from schools for rescue workers. All participants received a reimbursement for participation (10€/h) plus travel expenses.

**Psychometric measures**

The trait scale of the *German version of the State-Trait Anxiety Inventory* (STAI-T) is composed of 20 questions rated on a 4-point Likert scale ranging from 1 - nearly never to 4 - nearly always. Scores range from 20 to 80, with higher scores indicating greater trait anxiety. STAI-T has excellent internal reliability, good test-retest reliability, and high convergent validity in terms of correlations with parallel anxiety tests [1–3].

The German *version of the Center for Epidemiological Studies Depression Scale* (CES-D; German Allgemeine Depressions Skala, ADS) is a self-report instrument assessing the frequency of 20 depressive symptoms in the last week using a 4-point scale ranging from 0 - rare to 3 - mostly. The score ranges from 0 to 60. The ADS shows good internal and test-retest reliability, and high convergent validity [4,5].

The German version of the *Symptom Checklist-90-R* (SCL-90-R) consists of 90 items rated on a 5-point Likert scale ranging from 0 - not at all to 4 – very strong. The depression and anxiety subscales scores are calculated as the mean score and range from 0 to 4, and the scale shows high internal consistency and good convergent and divergent validity [6–8].

The *Childhood Trauma Questionnaire* (CTQ) is composed by 34 items rated on a 5-point Likert scale (1 = never true - 5 = very often true) that assesses the severity of adverse childhood experiences. The 5 subscales (emotional abuse, physical abuse, sexual abuse, emotional neglect, and physical neglect) have good test-retest reliability, and high internal consistency [9,10]. The CTQ also contains a denial scale that evaluates the likelihood of underreporting traumatic experiences. In the present study the sum score of the 5 subscales, not including the denial scale, has been used, that ranges from 5 to 125. We used the validated German version of the scale [11,12].

The *Trier Inventory for Chronic Stress* (TICS) is composed by 57 items assessing 9 chronic stressors: work and social overload, pressure to perform, work discontent, excessive demand at work, lack of social recognition, social tensions or isolation and chronic worrying. Each item is rated on a 5-point Likert scale (from 0 - never to 4 - very often) in respect to how often the subject had experienced a certain situation within the last 3 months. The internal consistency and test-retest reliability for this scale are good to very good [13,14].

**Statistical Analyses**

In case of violation of normality assessed by the Shapiro Wilk’s test, a logarithmic transformation was performed (Table S1).

**Supplementary results**

**Study participants**

Mean age, reported severity of childhood adversities, current stress load, anxiety trait and depressive symptoms did not significantly differ between men and women.

**Table S1**

**Data transformation**

Logarithmic transformation was performed to reduce the skewedness and curtosis of childhood adversities, depressive and anxiety symptoms measures distributions. [Abbreviations: SD - standard deviation; ADS - center for epidemiological studies depression scale; STAI-T – trait scale of the state and trait anxiety inventory; SCL-90-R – Symptoms Checklist-90-R].

|  | **Non-transformed** | | | **Log_10_ transformed** | | |
| --- | --- | --- | --- | --- | --- | --- |
|  | **Mean±SD** | **Skewedness** | **Curtosis** | **Mean±SD** | **Skewedness** | **Curtosis** |
| **Childhood adversities** | 31.635±9.759 | 1.334 | 1.506 | 1.483±0.121 | 0.725 | -0.020 |
| **Depressive symptoms**  **(ADS)** | 9.365±6.361 | 1.293 | 1.569 | 0.870±0.318 | -0.541 | 0.475 |
| **Anxiety symptoms**  **(STAI-T)** | 32.984±9.454 | 1.157 | 0.963 | 1.503±0.115 | 0.574 | -0.149 |
| **Depressive symptoms**  **(SCL-90-R)** | 0.319±0.514 | 2.697 | 8.282 | 0.098±0.129 | 1.762 | 3.072 |
| **Anxiety symptoms**  **(SCL-90-R)** | 0.205±0.281 | 2.431 | 6.815 | 0.072±0.085 | 1.738 | 3.410 |

**Table S2**

**Direct effects and correlation coefficients of the final and confirmatory models**

[Abbreviations: *MECP2* - methyl-CpG binding protein 2; b - unstandardized coefficient; s - covariance estimate; SE - standard error; β - standardized coefficient; r - Pearson’s correlation coefficient. Symbols: underlined - significant results].

| **Direct effects / Correlation coefficients** | **b/s (SE)** | **β/r** | **p-value** |
| --- | --- | --- | --- |
| ***Final Model*** |  |  |  |
| *MECP2**gender → childhood adversities | 0.036 (0.014) | 0.303 | 0.012 |
| childhood adversities → depressive symptoms | 0.712 (0.321) | 0.271 | 0.027 |
| childhood adversities → anxiety symptoms | 0.355 (0.112) | 0.373 | 0.002 |
| depressive symptoms ↔ anxiety symptoms | 0.015 (0.004) | 0.459 | <0.001 |
| ***Chronic stress model*** |  |  |  |
| *MECP2**gender → chronic stress | 0.101 (0.124) | 0.104 | 0.412 |
| chronic stress → depressive symptoms | 0.136 (0.037) | 0.426 | <0.001 |
| chronic stress → anxiety symptoms | 0.068 (0.012) | 0.588 | <0.001 |
| depressive symptoms ↔ anxiety symptoms | 0.01 (0.004) | 0.373 | 0.006 |
| ***Depression/anxiety scales subtitution model*** |  |  |  |
| *MECP2**gender → childhood adversities | 0.036 (0.014) | 0.303 | 0.012 |
| childhood adversities → depressive symptoms | 0.148 (0.087) | 0.300 | 0.013 |
| childhood adversities → anxiety symptoms | 0.32 (0.129) | 0.210 | 0.090 |
| depressive symptoms ↔ anxiety symptoms | 0.008 (0.002) | 0.825 | <0.001 |

**Figure S1**

**
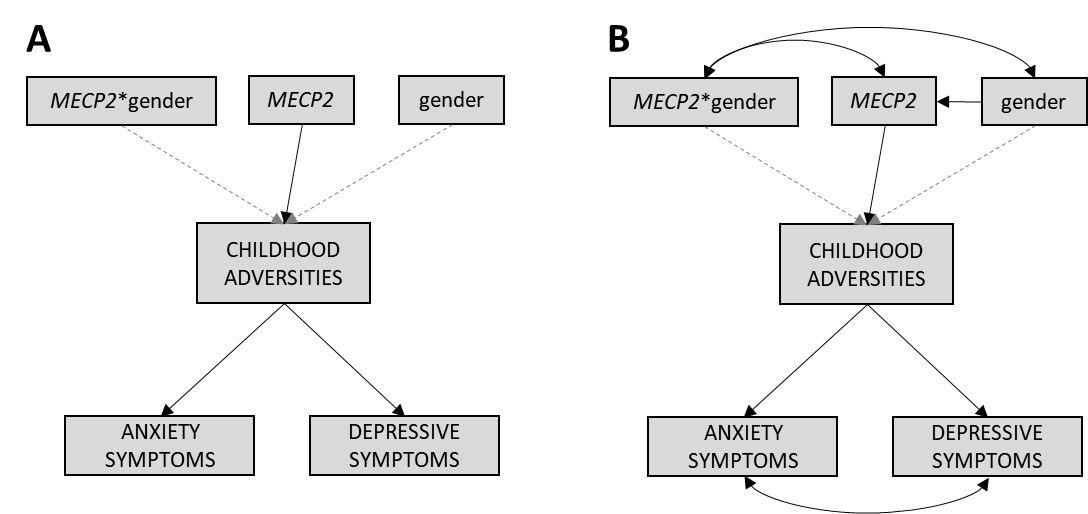
**

**Figure S1 – Initial hypothesized models.**

**A)** The initial model tested the hypothesis that *methyl-CpG binding protein 2 expression* and gender predicted alone and in interaction the severity of anxiety and depressive symptoms through the mediation of childhood adversities. **B)** Model respecifications were made by removing nonsignificant directed arcs and adding correlated paths as indicated by modification indices that were consistent with the hypothesis. [Symbols:
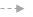
directed arcs (nonsignificant);
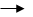
directed arcs (p<0.05);
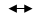
correlations (p<0.05)].

**Supplementary references**

1. Knight RG, Waal Manning HJ, Spears GF. Some norms and reliability data for the State-Trait Anxiety Inventory and the Zung Self-Rating Depression scale. Br J Clin Psychol. 1983;22:245–249.

2. Spielberger CD, Gorsuch RL, Lushene RE. STAI manual for the state-trait anxiety inventory. Self-Evaluation Questionnaire. Consulting Psychologists Press; 1970.

3. Laux L, Glanzmann P, Schaffner P, Spielberger C. Das State-Trait Angstinventar [The state-trait anxiety inventory]. Göttingen: Hogrefe; 1981.

4. Schmitt A, Hermanns N, Kulzer B, Gahr A, Haak T. Depressionsscreening mit der Allgemeinen Depressionsskala (ADS) bei Diabetespatienten im stationären Setting [Depression screening with the General Depression Scale (ADS) in diabetes patients in an inpatient setting]. Diabetol Und Stoffwechsel. 2013;9:77.

5. Hautzinger M, Bailer M. Allgemeine Depressions-Skala [General Depression-Scale]. Hogrefe; Göttingen; 1993.

6. Schmitz N, Hartkamp N, Kiuse J, Franke GH, Reister G, Tress W. The Symptom Check-List-90-R (SCL-90-R): a German validation study. Qual Life Res. 2000;9:185–193.

7. Koeter MWJ. Validity of the GHQ and SCL anxiety and depression scales: A comparative study. J Affect Disord. 1992;24:271–279.

8. Don Morgan C, Wiederman MW, Magnus RD. Discriminant validity of the SCL-90 dimensions of anxiety and depression. Assessment. 1998;5:197–201.

9. Bernstein DP, Stein JA, Newcomb MD, Walker E, Pogge D, Ahluvalia T, et al. Development and validation of a brief screening version of the Childhood Trauma Questionnaire. Child Abus Negl. 2003;27:169–190.

10. Bernstein DP, Fink L, Handelsman L, Foote J, Lovejoy M, Wenzel K, et al. Initial reliability and validity of a new retrospective measure of child abuse and neglect. Am J Psychiatry. 1994;151:1132–1136.

11. Wingenfeld K, Spitzer C, Mensebach C, Grabe H, Hill A, Gast U, et al. Die deutsche Version des Childhood Trauma Questionnaire (CTQ): Erste Befunde zu den psychometrischen Kennwerten [The German Version of the Childhood Trauma Questionnaire (CTQ): Preliminary Psychometric Properties]. Psychother Psychosom Med Psychol. 2010;60:442–450.

12. Bader K, Hänny C, Schäfer V, Neuckel A, Kuhl C. Childhood Trauma Questionnaire - Psychometrische Eigenschaften einer deutschsprachigen Version [Psychometric Properties of a German Language Version of the Childhood Trauma Questionnaire]. Z Klin Psychol Psychother. 2009;38:223–230.

13. Petrowski K, Paul S, Albani C, Brähler E. Factor structure and psychometric properties of the Trier inventory for chronic stress (TICS) in a representative German sample. BMC Med Res Methodol. 2012;12:42.

14. Schultz P, Schlotz W. Trierer Inventar zur Erfassung von chronischem Stress (TICS): Skalenkonstruktion, teststatistische Überprüfung und Validierung der Skala Arbeitsüberlastung [The Trier Inventory for the Assessment of Chronic Stress (TICS)]. Diagnostica. 1999;45:8–19.
